# Modelling the cost-effectiveness of introducing the RTS,S malaria vaccine relative to scaling up other malaria interventions in sub-Saharan Africa

**DOI:** 10.1136/bmjgh-2016-000090

**Published:** 2017-01-24

**Authors:** Peter Winskill, Patrick GT Walker, Jamie T Griffin, Azra C Ghani

**Affiliations:** 1 Department of Infectious Disease Epidemiology, MRC Centre for Outbreak Analysis and Modelling, Imperial College London, London, UK; 2 School of Mathematical Sciences, Queen Mary University of London, London, UK

## Abstract

**Objectives:**

To evaluate the relative cost-effectiveness of introducing the RTS,S malaria vaccine in sub-Saharan Africa compared with further scale-up of existing interventions.

**Design:**

A mathematical modelling and cost-effectiveness study.

**Setting:**

Sub-Saharan Africa.

**Participants:**

People of all ages.

**Interventions:**

The analysis considers the introduction and scale-up of the RTS,S malaria vaccine and the scale-up of long-lasting insecticide-treated bed nets (LLINs), indoor residual spraying (IRS) and seasonal malaria chemoprevention (SMC).

**Main outcome measure:**

The number of *Plasmodium falciparum* cases averted in all age groups over a 10-year period.

**Results:**

Assuming access to treatment remains constant, increasing coverage of LLINs was consistently the most cost-effective intervention across a range of transmission settings and was found to occur early in the cost-effectiveness scale-up pathway. IRS, RTS,S and SMC entered the cost-effective pathway once LLIN coverage had been maximised. If non-linear production functions are included to capture the cost of reaching very high coverage, the resulting pathways become more complex and result in selection of multiple interventions.

**Conclusions:**

RTS,S was consistently implemented later in the cost-effectiveness pathway than the LLINs, IRS and SMC but was still of value as a fourth intervention in many settings to reduce burden to the levels set out in the international goals.

**Key questions**

**What is already known about this topic?**

- We sought to evaluate the relative cost-effectiveness of introducing the RTS,S malaria vaccine in sub-Saharan Africa compared with further scale-up of existing interventions. We did not identify any studies modelling the impact and costs of intervention packages with respect to scaling-up coverage.

**What are the new findings?**

- We find that implementing the RTS,S malaria vaccine generally only enters the optimal pathway of scale-up of interventions once very high coverage of vector control interventions, along with seasonal malaria chemoprevention in settings where it is recommended, has been achieved.

**Recommendations for policy**

- While the RTS,S malaria vaccine can be an effective tool for reducing burden, enhancing coverage of vector control should generally remain of higher priority across sub-Saharan Africa. This is particularly the case within settings where universal coverage of long-lasting insecticide-treated nets has not yet been achieved.

## Introduction

Following the adoption of the Millennium Development Goals (MDGs) in 2000, the concurrent targets of increasing the proportion of children under 5 years that sleep under a bed net and that have access to appropriate antimalarial drugs^1^ have been associated with an estimated 37% decline in malaria case incidence and 60% decline in malaria mortality.^2^ ^3^ These gains have been achieved with substantial investment in malaria which has more than doubled over the last decade, from less than $1 billion in 2005 to over $2.5 billion in 2015.^2^ However, since 2011, the acceleration in funding has slowed, plateauing in recent years.^2^ Funding gaps therefore remain an inevitable issue for future control and elimination efforts and thus optimising the use of available resources is paramount. Within this context, new interventions must be evaluated not only on their direct cost-effectiveness, but comparative to the other intervention options.

In early 2015, the final results from the large phase III trial of the RTS,S malaria vaccine across 11 sites in Africa were published.^4^ Reported efficacy against clinical and severe malaria disease in the presence of high use of bed nets for prevention and alongside a high level of access to care was moderate. The trial reported 36.3% (95% CI 31.8% to 40.5%) efficacy against clinical disease in children aged 5–17 months over 4 years under a four-dose schedule and 32.2% (13.7% to 46.9%) efficacy against severe disease.^4^ Nevertheless, in the high transmission sites contributing most of the disease episodes, there was a significant public health impact, with between 1000 and 6000 cases estimated per 1000 population over 4 years of follow-up in the six higher transmission sites.^4^ To estimate the wider public health impact and cost-effectiveness of the vaccine in settings representative of current levels of ongoing malaria transmission, a WHO working group was formed to compare the outputs from four mathematical models parameterised using the trial data. The results from this comparison demonstrated that the vaccine could have a substantial public health impact across settings ranging from 10% to 60% parasite prevalence in 2–10 year olds.^5^ Furthermore, assuming a midrange cost of $5 per dose under a four-dose schedule, the vaccine was considered to be highly cost-effective (incremental cost-effectiveness ratio (ICER) of $44–$279 per disability-adjusted life year (DALY)) within these same transmission levels.^5^

Existing malaria interventions are also highly cost-effective.^6^ Although there are substantial differences between the methodologies used to make these estimates, a 2011 study estimated the median ICER per DALY at $27 (range $8.15–110) for long-lasting insecticide-treated nets (LLINs) and $143 (range $135–150) for indoor residual spraying (IRS), while for seasonal malaria chemoprevention (SMC), the cost per case averted has been estimated at $68 (95% CI $62 to $75), similar to comparable metrics for LLINs and IRS.^7^ Thus, in areas in which coverage of these interventions is not yet universal, it is important to understand the relative cost-effectiveness of the full suite of interventions and where the RTS,S malaria vaccine could contribute. Importantly, this needs to take into account the diminishing marginal returns associated with the scale-up of interventions that may lead to a higher unit cost at high levels of coverage.^8^ ^9^

Here, we use a well-established transmission model for *Plasmodium falciparum* malaria and its associated interventions^10^ to estimate the cost and impact of different intervention packages at varying levels of scale-up. We evaluate these packages over a wide range of transmission settings and use the estimates to derive the most cost-effective pathways for scaling-up malaria interventions in order to inform decisions about the introduction of the RTS,S malaria vaccine.

## Methods

### Transmission and intervention model

We used an established model of *P. falciparum* malaria transmission that incorporates the full suite of interventions.^10^ In brief, individuals that are initially susceptible are exposed to infection with *P. falciparum* malaria from bites of infectious mosquitoes. The rate at which susceptible individuals become infected is influenced by mosquito density and infectivity and is moderated by immunity. Infants are partially protected for the first 6 months of their life by passively acquired maternal immunity and individuals acquire natural immunity with repeated exposures through time. Infected individuals may be treated, after which follows a period of prophylaxis before returning to the susceptible class. Infected individuals can develop clinical disease,^11^ which may progress to severe disease and possibly death.^12^ A proportion of infected individuals harbour asymptomatic infections, some of which may also be subpatent. The model explicitly incorporates mosquito-population dynamics,^13^ allowing the modelling of various protective, repellent and killing aspects of the vector-control interventions. We adopt the same methodology for incorporating the RTS,S vaccine into the model as used for a large model-comparison exercise.^5^ The RTS,S vaccine model dynamics use a biphasic antibody decay model fitted to the phase III individual-level trial data, allowing the level of protection against clinical disease to be captured with respect to antibody titre postvaccination.^14^

### Transmission settings

To capture the range of transmission settings across Africa, we generated a baseline set of ‘strata’. These were characterised by the parasite prevalence in the absence of interventions other than treatment, the annual seasonal pattern of transmission and the mosquito vector species present and their associated bionomics (which affect the predicted impact of LLINs and IRS). The mean parasite prevalence over the full year in 2–10 year-olds (*Pf*PR_2_10_) was simulated in 17 bands between 0.1% and 80% to capture the range of transmission levels observed prior to intervention scale-up.^3^ Four seasonality profiles were simulated based on Fourier-transformed average rainfall patterns obtained from satellite data between 2003 and 2006:^15^ (A) highly seasonal—with a single strong peak in rainfall characteristic of the Sahel region; (B) seasonal—with a less strong peak in rainfall characteristic of West Africa coastal areas; (C) bimodal—with one large and a second smaller peak in rainfall characteristic of East and Southern Africa; and (D) non-seasonal characteristic of perennial levels of rainfall observed in Central Africa. The four mosquito vector profiles capture behavioural difference in the levels of anthropophagy (the human biting index), endophily (indoor biting), endophagy (indoor resting) and the timing of bites relative to sleeping hours. Rather than simulating species and their combinations, these profiles represent a range of vector species bionomics moving from behaviours associated with *Anopheles gambiae s.s.*/*Anopheles funestus* to those associated with *Anopheles arabiensis*. The combination of parasite prevalence bands, seasonality of transmission and vector bionomics resulted in 272 baseline strata. Further details are provided in the online supplementary materials.

10.1136/bmjgh-2016-000090.supp1 supplementary materials

### Interventions

For each strata, we simulated the impact of all possible applicable combinations from a set of four interventions (LLINs, IRS, SMC and RTS,S) at a range of coverage levels (see online supplementary materials for details), resulting in a total of 306 000 simulations. Throughout we assumed that an LLIN would cover 1.8 people (consistent with the approach taken in the World Malaria Report^2^) and that nets would be distributed on a 3-yearly cycle. The bed net model further captures loss of adherence, decay in insecticides and wear-and-tear over time^16^ and we define coverage of bed nets as usage as reported in DHS/MIS surveys.^17^ ^18^ For IRS, we assumed that a DDT-like insecticide was used and applied once a year. For this intervention, coverage was defined as the proportion of the population residing in a house that was sprayed in the previous year. SMC was simulated following WHO recommendations to children between 6 months and 5 years of age, with 3 monthly doses of SP-amodiaquine in seasonal settings, with the second dose timed to occur at the seasonal peak in transmission.^19^ Coverage was defined as the proportion of eligible children who received all three doses and we did not model the effect of partial doses. Following the recent WHO recommendations, we considered a four-dose vaccine schedule in children aged 5–27 months. We assumed children would be vaccinated at 6, 7.5 and 9 and 27 months, with the timings of the first doses chosen to coincide with other contacts with healthcare at 6 and 9 months. Coverage was defined as the proportion of eligible children receiving the full four doses. A 20% drop off between those receiving the first three does and the fourth dose was included to capture loss of follow-up.^5^

Receipt of a single intervention was assumed to be correlated across the population. When two or more interventions were included, we explored two options: no correlation in receipt (ie, independent random distribution of both interventions) or full correlation (ie, those that receive the first intervention are will also receive the second). Throughout we assumed that 60% of those with clinical disease received prompt and effective first-line treatment.

### Costing

In the absence of detailed country-level data for all interventions, we adopted a unit costing approach. These were derived from the literature and inflated to 2015 US$ (table 1).

**Table 1:** Unit cost values for interventions

Intervention	Unit cost (2015 US $)	Reference/notes
LLINs	7.03 per LLIN delivered	White *et al*.^20^ The 2009 costing was not inflated as more recent estimates are similar^21^
IRS	5.41 per person protected	PMI AIRS project^22^
SMC	1.65 per child per round	CHAI/MSF estimates^23^ ^24^
RTS,S	39.25 per fully vaccinated child	Under the assumption of $5 per dose^5^ ^25^
Treatment (clinical cases)	2.59 per person (test+treatment)	26
Treatment (severe cases)	33.54 per person	20

We considered two approaches for costing increasing coverage of the four interventions. The first approach assumed increases in coverage were associated with linear increases in cost, while in the second approach, we derived non-linear relationships between coverage and unit costs. For this second approach, the number of nets required to achieve a given coverage level (defined by usage) was obtained from Bhatt *et al*,^27^ assuming a net-retention half-life of 3 years and the business-as-usual net allocation process. We estimated similar health production functions for IRS, SMC and the vaccine by fitting a model to data relating the cost and coverage of these interventions in a Bayesian framework (see online supplementary materials for full details). The total cost (P) of delivering an intervention to an individual is assumed to consist of two components: the commodity cost (U) and the delivery cost (D)
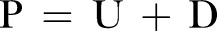


The commodity cost remains fixed per person (under the assumption that economies of scale have been reached) with respect to coverage (C). The delivery cost per person is fixed at a baseline amount, N, until coverage reaches a given threshold, C_τ_, above which the delivery costs increase logarithmically
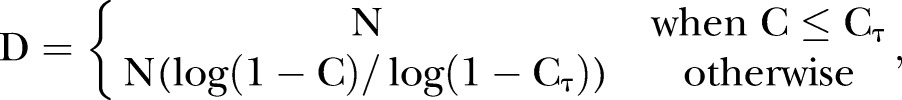


### Outcome measures

We considered a number of outcome measures to evaluate the incremental cost-effectiveness of each intervention. The primary outcome measure presented throughout is the number of cases averted over a 10-year period. Other outcome measures considered include the DALYs averted and the number of cases averted in children aged 6 months–5 years old and over a 10-year period (see online supplementary materials for more details on calculation).

### Estimating cost-effective scale-up

**Figure 1:**
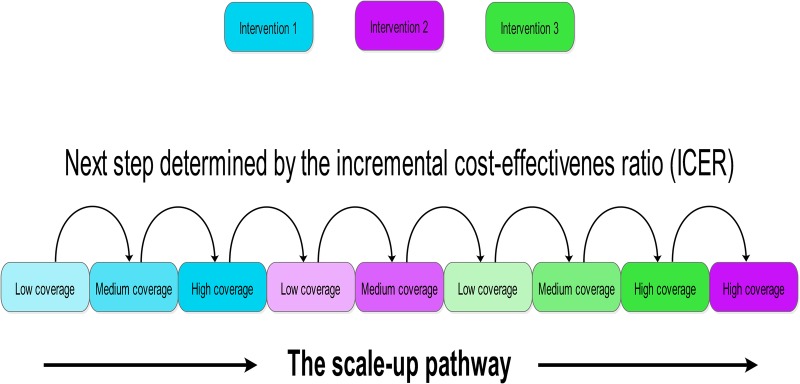
Schematic of the cost-effective scale-up pathway. For each transmission strata, the cost-effective scale-up of interventions was estimated, starting with no intervention coverage then scaling up coverage based on the most favourable incremental cost-effectiveness ratio. Scale-up ceased when all interventions were at full coverage or elimination had occurred.

For each transmission strata, the cost-effective scale-up of interventions was estimated using the following steps: (i) start with 0% usage/coverage of all interventions, (ii) for each available intervention, calculate the ICER of scaling up to the next usage/coverage level, (iii) implement the step with the lowest associated ICER (the most cost-effective), (iv) repeat the process (i–iii) until the scale-up of all interventions has been maximised or elimination is achieved. This process is summarised in figure 1. It should be noted that while this approach results in the most cost-effective next level of intervention coverage, the resulting intervention packages are not necessarily maximally efficient for a given budget since this reflects a gradient descent optimisation (stepwise) rather than a multidimensional optimisation approach. Nevertheless, it is used here to illustrate the pathways at each point in the absence of defined budget limits.

### Cost sensitivity analysis

We assessed the sensitivity of the order of scale-up to uncertainty in the costs of interventions and their associated production functions. The analysis was repeated with 100 random draws from the posterior predictive interval for the IRS, SMC and RTS,S production functions. LLIN costs were randomly drawn from the interval between the least optimistic (net retention half-life of 2 years and current allocation process) and most optimistic (net retention half-life of 3 years and improved allocation process) net-allocation models.^27^ For each 100 runs and across all transmission strata, the relative occurrence of each intervention at each scale-up step was measured.

The sensitivity of the scale-up order to the assumed $5 per dose cost of the vaccine was also examined by incrementally decreasing its price (increases were not included, given the initial results). At each step, the proportion of settings where the vaccine appeared in the pathway before LLINs, IRS and SMC was then recorded.

## Results

**Figure 2:**
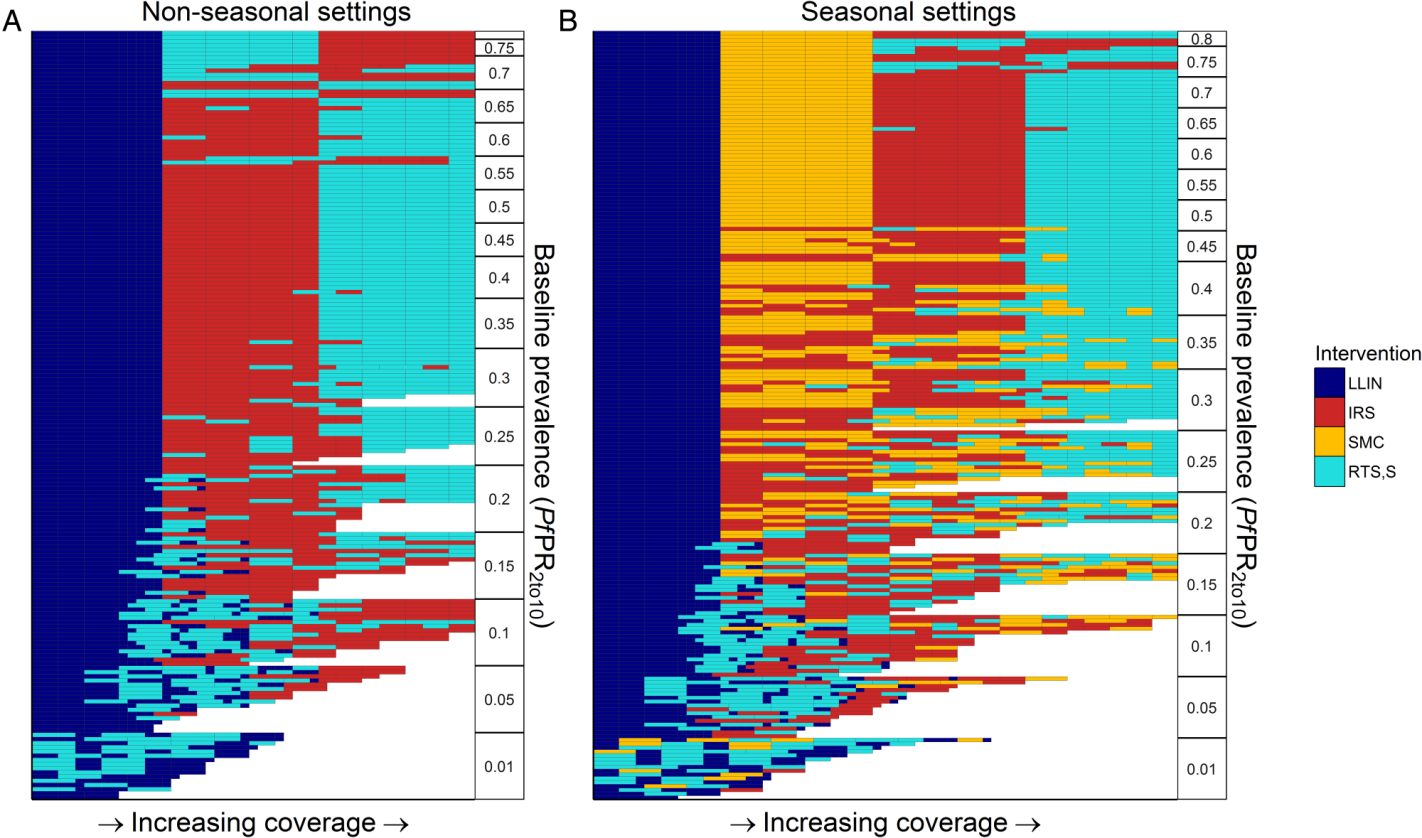
Costs-effective scale-up pathways with linear costs. Each row represents a cost-effective scale-up pathway for a specific transmission setting (baseline *Pf*PR_2_10_, seasonal profile, vector profile, intervention correlation) ordered by *Pf*PR_2_10_ on the y-axis. Interventions are scaled-up in the order reading along the row from left to right, with the fill colour representing the intervention being scaled-up. Panels split the output into (A) non-seasonal settings and (B) seasonal settings, with the latter including seasonal malaria chemoprevention as an option.

Figure 2 shows the scale-up pathways for the combination of the four interventions across a range of transmission levels characterised by their baseline *Pf*PR_2–10_ (in the absence of interventions other than treatment of clinical cases). For the majority of settings, across the full-range of baseline *Pf*PR_2–10_, for vector bionomics characteristic of the three main species found in Africa (*A. gambiae s.s.*, *A. arabiensis* and *A. funestus*) and for seasonal and non-seasonal settings, LLINs appear first in the most cost-effective pathway. At baseline *Pf*PR_2–10_ <5%, LLINs and RTS,S are predicted to reduce ongoing transmission to pre-elimination levels. If scale-up of these interventions is unable to achieve pre-elimination transmission levels, our results suggest that LLINs should be scaled up to very high usage levels (75%) prior to introducing additional interventions if cost-effectiveness is the single deciding factor. After this level has been achieved, in non-seasonal settings, IRS is the second-most cost-effective intervention at lower baseline *Pf*PR_2–10_ (5%< *Pf*PR_2–10_ <65%), while RTS,S is estimated to be the second most cost-effective intervention in settings with baseline *Pf*PR_2–10_ ≥65%. In seasonal settings, SMC is generally the second most cost-effective intervention to introduce prior to IRS or RTS,S.

**Figure 3:**
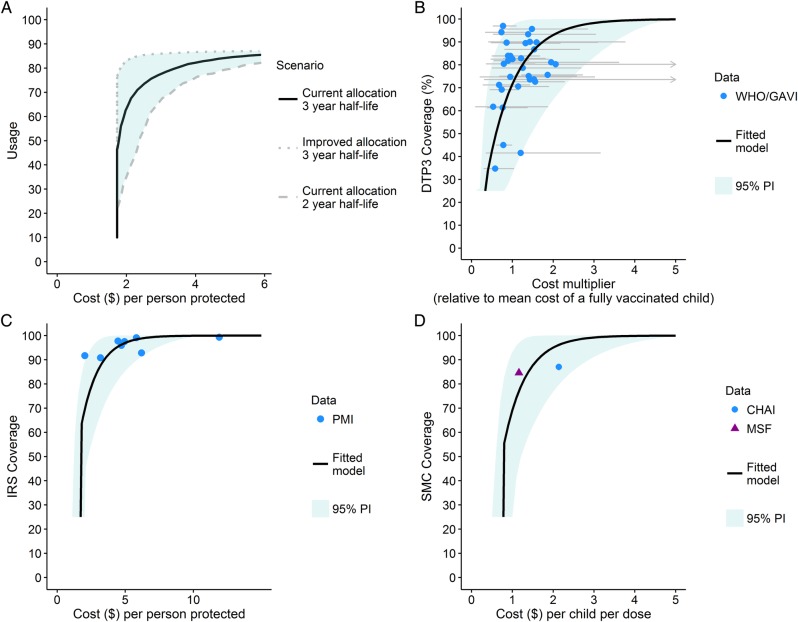
Non-linear production functions. Production functions estimate the non-linear relationships between intervention usage or coverage and the cost per person. (A) Cost per person protected by long-lasting insecticide-treated nets, taken from Bhatt *et al*.^27^ Three scenarios: assuming current allocation model and a 3-year net-retention half-life, an improved allocation model and 3-year net retention half-life and a current allocation model and 2-year net retention half-life are shown by the solid black, dotted grey and dashed grey lines, respectively. (B) DTP3 coverage as a function of the price per person (standardised by the cost of a fully vaccinated child). (C) IRS coverage as a function of the cost per person protected based on President's Malaria Initiative data.^22^ (D) Seasonal malaria chemoprevention coverage as a function of the costs per child per dose based on Clinton Health Access Initiative^23^ and Medicins San Frontiers estimates.^24^ For (B–D), black lines represent the best-fit model and shaded areas the 95% prediction interval.

The results shown in figure 2 assume a single unit cost that does not change with increasing coverage. However, as higher coverage levels are sought, costs tend to increase as it becomes more difficult to fill the remaining coverage gaps and to access the hardest-to-reach populations. Figure 3 shows our estimated empirical production functions for IRS, SMC and vaccination (based on DTP3 data) alongside the previously published estimated for LLIN usage.^27^ All four functions have a similar shape, with increasing costs at high coverage. However, for IRS and SMC, the recorded coverage was consistently high (>80%) and the limited number of data points mean that this function is uncertain.

**Figure 4:**
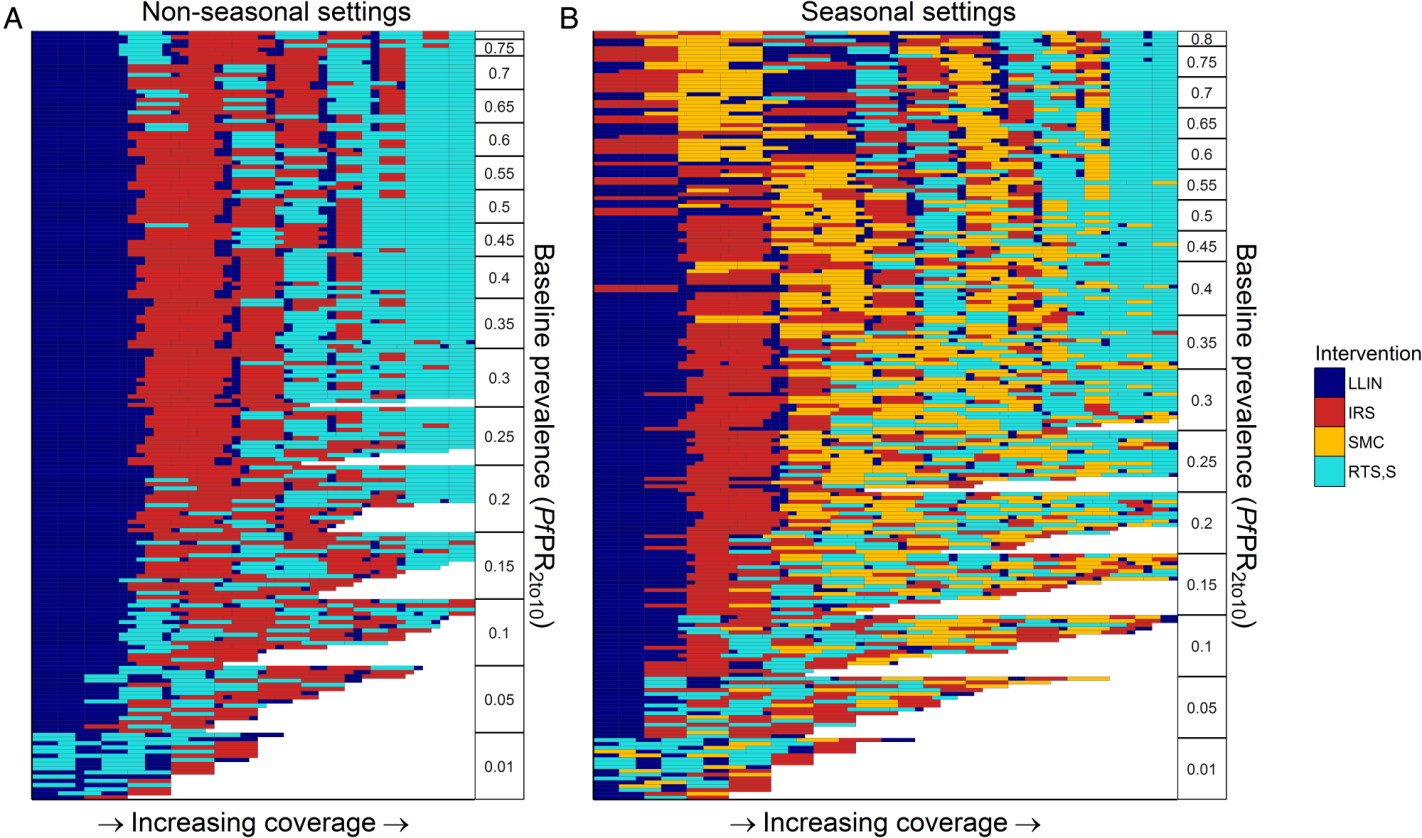
Costs-effective scale-up pathways with non-linear costs. Each row represents a cost-effective scale-up pathway for a specific transmission setting (baseline *Pf*PR_2_10_, seasonal profile, vector profile, intervention correlation) ordered by *Pf*PR_2_10_ on the y-axis. Interventions are scaled-up in the order reading along the row from left to right, the fill colour representing the intervention being scaled-up. Panels split the output into (A) non-seasonal settings and (B) seasonal settings, with the latter including seasonal malaria chemoprevention as an option.

Including the non-linear production functions shown in figure 3 leads to a substantially more complicated pattern of scale-up pathways (figure 4). With these additional non-linearities, alternative interventions are always introduced before LLIN usage is increased to the maximum level due to the high estimated cost of achieving high LLIN usage. In non-seasonal settings, LLINs are estimated to be the most cost-effective initial interventions. Across the majority of settings with baseline 5%<*Pf*PR_2–10_<60%, IRS appears second with RTS,S also introduced once moderate levels of IRS coverage have been achieved. In seasonal settings, for baseline *Pf*PR_2–10_<50%, LLINs remain the first most cost-effective intervention. However, in settings with higher baseline transmission, SMC and RTS,S appear earlier. Following this, the second and third most cost-effective additional interventions vary by setting, with IRS being favoured in locations where the vector bionomics are more amenable to insecticidal control, whereas RTS,S or SMC are favoured in settings with less favourable vector bionomics.

**Figure 5:**
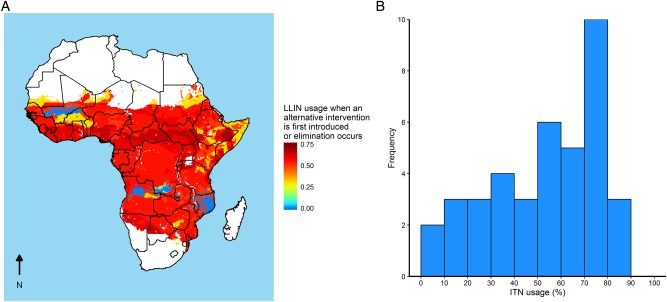
Long-lasting insecticide-treated net (LLIN) primary scale-up and usage statistics. (A) The LLIN usage at which an alternative intervention is first introduced for sub-Saharan Africa. For much of sub-Saharan Africa, LLIN scale-up to medium or high usage levels before any other intervention is implemented is the most cost-effective. In seasonal areas, indoor residual spraying or seasonal malaria chemoprevention can be the first most cost-effective intervention. (B) The distribution of country level LLIN usage estimates for 2015.^3^

Figure 5 illustrates the translation of these generic results to locations in sub-Saharan Africa using estimates of vector species presence, baseline *Pf*PR_2–10_ and seasonality. Across the majority of settings, LLINs are the first most cost-effective intervention. Using the results from figure 4, the LLIN usage at which a second intervention is estimated to be more cost-effective than further LLIN scale-up is shown in figure 4A. In the majority of settings, we estimate a switch is cost-effective at 55–65% LLIN usage, although in some seasonal areas in West Africa and in areas with high estimated baseline *Pf*PR_2–10_, other interventions are estimated to be more cost-effective at lower levels of LLIN usage. This threshold is similar to the levels of usage that have now been achieved in many parts of Africa (figure 5B).

**Figure 6:**
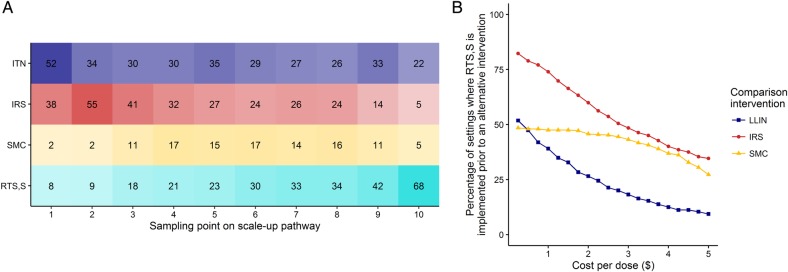
Sensitivity of the results to variations in costs of the interventions. (A) The sensitivity of the scale-up pathway to uncertainties in the health production functions determining the cost of each intervention. Columns represent 10 equally spaced samples (from left to right) along the scale-up pathway. Numbered cells denote the number of instances, out of 100 realisations, that a given intervention was implemented at that step. (B) Sensitivity of outcome to assumed cost per dose of RTS,S. The assumed cost per dose was decreased in incremental amounts. At each step, the proportion of settings in which the RTS,S was implemented before either long-lasting insecticide-treated nets, indoor residual spraying or seasonal malaria chemoprevention (blue, red and yellow lines, respectively) is shown.

While the results vary for different assumed unit costs for each intervention, in a sensitivity analysis of these costs, we found that the order of scale-up is generally maintained (figure 6A). Since the price of the RTS,S vaccine has not been released, we additionally assessed the sensitivity to the assumed price per dose. Figure 6B shows the proportion of scenarios in which RTS,S, at a given price per dose, is estimated to occur before other interventions in the scale-up pathway. In general, RTS,S remains late in the pathway. However, this pattern changes if the price per dose drops below US$3 where the relative cost-effectiveness becomes comparable to IRS and SMC. However, even at a very low cost per dose (<US$1.00), LLINs are estimated to remain a more cost-effective intervention than RTS,S in approximately half of the settings.

## Discussion

Our analysis demonstrates that LLINs remain the most cost-effective first intervention to reduce malaria transmission across the broad range of transmission settings observed in sub-Saharan Africa. The high consumption and competitive market for LLINs has driven costs down over the last 10 years.^21^ This, coupled with their dual effect of personal-level and community-level protection,^28^ makes them highly cost-effective. This finding was consistent for all outcome measures considered (see online supplementary materials S1–S4). Furthermore, our results indicate that, based on cost-effectiveness considerations, the RTS,S vaccine should be considered a secondary intervention alongside the two other WHO-recommended malaria interventions for this region—SMC and IRS. The recommended schedule for the RTS,S vaccine that will be tested in pilot implementation is for four doses given in children aged 5–27 months, who constitute a small subset of the exposed population. The vaccine offers partial protection to this group over a duration of ∼4 years.^4^ ^14^ As a result, this vaccine does not have the benefit of inducing herd-immunity in the population and is considerably more expensive per person than the other interventions considered here (at the assumed cost of $5 per dose), lowering its relative cost-effectiveness. Thus, other than in high transmission settings where there is a high burden of disease in young children, we find that RTS,S enters the cost-effectiveness scale-up pathway later on when other potential options for reducing transmission and/or protecting from disease are already maximised.

While increasing usage of LLINs is identified as the most cost-effective first intervention, once these levels reach 50–60%, we estimate that the three alternative interventions—IRS, SMC and RTS,S—become increasingly competitive when comparing the relative cost-effectiveness. This level of LLIN usage is similar to the levels reported for many countries in sub-Saharan Africa in 2015,^3^ suggesting that context-specific cost-effectiveness considerations may become increasingly important as investment in current or new interventions are considered. The inclusion of IRS as a secondary vector control option could further reduce onward transmission through providing additional protection for those that do not consistently use nets or over the periods between net distribution rounds in which the integrity of the net and/or efficacy of the insecticide has decayed. However, the trial data on the combination of these two vector control interventions remain inconclusive^29^ ^30^ and hence close monitoring would be required to fully understand the operational impact of their combined use. Furthermore, any recommendations in favour of vector control must be made in the light of current and potential future insecticide resistance, the effects of which were not included in this analysis. In a small number of settings, characterised by a high level of seasonal transmission and intense transmission, we identified either SMC or IRS as the most cost-effective first intervention. SMC and IRS are temporally targeted at the peak transmission season, and this therefore increases their cost-effectiveness relative to non-seasonally targeted interventions.^31^ A comparison of the impact of pairwise combinations of interventions is included in online supplementary material S5.

We explicitly excluded treatment scale-up options from the cost-effectiveness analysis for several reasons. First, treatment has been previously shown to be highly cost-effective.^6^ Therefore, equitable access to treatment for severe disease is an ethical priority and universal scale-up of treatment coverage is important to preserve. Second, while increasing treatment coverage, and therefore costs, affects the absolute cost-effectiveness of other interventions (with lower treatment coverage making them more cost-effective), the relative cost-effectiveness when comparing interventions (and therefore scale-up pathways) will be influenced far less. Third, the ability of a country to increase coverage of treatment will depend critically on health system capacity and hence vary geographically. Thus, a simple unit cost approach is unlikely to be appropriate. For other interventions, the commodities (particularly LLINs) are purchased through Global Fund pathways for which there is a coordinated tendering process. We have therefore adopted a unit costing approach for the interventions considered in this broad-scale comparison.

Systems-level inefficiencies,^32^ overallocation^27^ and systematic under-representation in hard-to-reach populations^33^ ^34^ may all contribute to diminishing marginal returns when investing in increasing the coverage of an intervention to very high levels. Our results demonstrate that it is important to capture these non-linearities when considering the relative costs-effectiveness of introducing new interventions such as the RTS,S malaria vaccine. With the simple assumption that costs associated with increasing the usage or coverage of an intervention increases linearly, we observe a very clear picture of cost-effective scale-up, with LLIN usage increased to the maximum level (75%) in nearly all settings before any other intervention is implemented. However, the inclusion of non-linear productions functions that capture the increasing cost associated with achieving high levels of coverage of any given intervention leads to a more complicated picture of the cost-effective scale-up pathway. While there was considerable uncertainty in our estimated production functions for each intervention, general patterns in scale-up remained fairly robust to this. However, further data on these patterns are critical to inform local planning. Subtle differences in the inflection points of the production functions affect when a switch between interventions is made. This is especially of note for the SMC production function where lack of data lead to considerable uncertainties in the resultant production function.

While cost-based uncertainties were explored in this analysis, we did not additionally explore the uncertainty in model structure or parameterisation due to the computational complexity in undertaking such an analysis. Clearly, model parameters and structures could affect the relative impact of interventions as well as the combinations of interventions needed to reach pre-elimination levels. Determining the cost-effective scale-up in a stepwise manner informed by the ICER always chooses the next most cost-effective option. This is analogous to a gradual scale-up of interventions over time, where future options are considered, given an established intervention landscape. However, for a given spend, the resulting intervention package estimated from a stepwise approach may differ from a global optima if all scale-up options were considered in unison.

Context-specific challenges to scaling-up a given intervention will always be present and cannot be represented in this style of analysis. To this end, policy decisions must take into account such challenges when considering recommendations in a specific setting. While a number of countries have achieved the high levels of LLIN usage that our analysis suggests to be cost-effective,^3^ in other settings, there may be barriers to achieving and sustaining the levels in the long term, nuancing the decision to target LLIN scale-up. Nevertheless, the ambitious targets set by the WHO for universal coverage may be integral in driving trends in net usage upwards, even if the target cannot be reached.
