# Supplementary material for: Modelling the cost-effectiveness of introducing the RTS,S malaria vaccine relative to scaling up other malaria interventions in sub-Saharan Africa
Source: BMJ Glob Health. 2017 Jan 24;2(1):e000090. doi: 10.1136/bmjgh-2016-000090 (PMC5321383; doi:10.1136/bmjgh-2016-000090)
Supplement: supplementary materials [file bmjgh-2016-000090supp.pdf]

Supplementary materials

Transmission model

Human Dynamics

We outline the model here in a deterministic formulation. For all simulations presented the equivalent individual-based stochastic model was used.[1] The stochastic version only differs structurally from the deterministic version in the non-exponential durations of prophylactic protection after treatment.

Let Λ be the force of infection, which is dependent upon the entomological inoculation rate (EIR) and determines the rate at which susceptible individuals (S) become infected. Infected individuals undergo a latent period (12 days) after which they may develop symptoms with a given probability, ϕ, or develop an asymptomatic infection (with probability $1-\phi$) and move to compartment A. Individuals who develop symptoms may either be successfully treated (with probability f_T, moving to state T or remain untreated or fail treatment (with probability $1-f_T$), moving to state D. An individual who has been successfully treated moves to state P, representing a period of prophylactic protection from re-infection, before returning to the susceptible state. Those failing treatment are assumed to become patently asymptomatic, moving to state A. Patent asymptomatic infections progress to sub-patent asymptomatic infections (state U) which are cleared at a given rate, returning an individual to the susceptible state. Super-infection may occur from states A and U.

These dynamics are captured by the following differential equations:

$$
\begin{aligned}
\frac{\partial S}{\partial t}+\frac{\partial S}{\partial a}+\frac{\partial S}{\partial \zeta} &= -\Lambda S + P/d_P + U/d_U \\
\frac{\partial T}{\partial t}+\frac{\partial T}{\partial a}+\frac{\partial T}{\partial \zeta} &= \phi f_T \Lambda (S+A+U) - T/d_T \\
\frac{\partial D}{\partial t}+\frac{\partial D}{\partial a}+\frac{\partial D}{\partial \zeta} &= \phi (1-f_T) \Lambda (S+A+U) - D/d_D \\
\frac{\partial A}{\partial t}+\frac{\partial A}{\partial a}+\frac{\partial A}{\partial \zeta} &= (1-\phi)\Lambda (S+U) + D/d_D - \phi \Lambda A - A/d_A \\
\frac{\partial U}{\partial t}+\frac{\partial U}{\partial a}+\frac{\partial U}{\partial \zeta} &= A/d_A - U/d_U - \Lambda U \\
\frac{\partial P}{\partial t}+\frac{\partial P}{\partial a}+\frac{\partial P}{\partial \zeta} &= T/d_T - P/d_P
\end{aligned}
\tag{1.1}
$$

where t is time, a age and ζ the rate at which an individual is bitten, d_T, d_D, d_A, d_U, d_P are the mean duration spent in states T, D, A, U, and P respectively.

Heterogeneity to exposure captures age-dependent exposure as well has heterogeneities in exposure due to other factors such as locational differences. Each individual is assigned a relative biting rate, ζ, parametrised with scale $=-\sigma^2/2$ and shape $=\sigma$ ensuring ζ has a mean of 1. The EIR and force of infection at age a are therefore:

$$
\begin{aligned}
\varepsilon &= \varepsilon_0 \zeta (1-\rho \exp(-a/a_0)) \\
\Lambda &= \varepsilon b
\end{aligned}
\tag{1.2}
$$

where ε_0 is the mean EIR for adults, b is the probability of infection upon being bitten by an infectious mosquito and ρ and a_0 parameterise the change in the rate of being bitten as a function of age.

The effects of immunity are included in the transmission model at a number of stages. These are:

i) Maternal immunity – A degree of protection against infection after being born is conferred from mother to child. This protection decreases the probability of infection upon being bitten by an infectious mosquito (b). The level of protection is a function of the mother's immunity and wanes at a given rate.

ii) Blood stage immunity – Reduces the probability of developing clinical disease upon infection (ϕ). Reduces blood stage parasite densities, leading to decreased detectability and decreased onwards infection of mosquitoes.

iii) Pre-erythrocytic immunity – Reduces the probability of infection upon being bitten by an infectious mosquito (b) in older children and adults.

Full details of the functional implementation of immunity in the transmission model are found in Griffin *et al* (2014).[2] Human parameters are shown in Table 1.

Table 1. Human transmission model parameters, definition and values.

Parameter	Description	Estimate (95% credible interval)
**Human infection duration**		
d_E	Latent period	12 days (fixed)
d_I	Patent infection	200 days (fixed)
d_T	Clinical disease (treated)	5 days (fixed)
d_D	Clinical disease (untreated)	5 days (fixed)
d_U	Sub-patent infection	110 days (87,131)
d_P	Prophylaxis following treatment	Drug-dependent
**Infectiousness to mosquitoes**		
t_l	Lag from parasites to infectious gametocytes	12.5 days (fixed)
c_D	Untreated disease	0.068 day^{-1} (0.039, 0.122)
c_T	Treated disease	Drug-dependent
c_U	Sub-patent infection	0.0062 day^{-1} (0.00056, 0.018)
θ	Relates probability of detection to infectiousness for asymptomatic infection	1.82 (0.603, 8.54)
**Age and heterogeneity**		
ρ	Age-dependent biting parameter	0.85 (fixed)
a_0	Age-dependent biting parameter	8 years (fixed)
σ^2	Variance of the log heterogeneity in biting rates	1.67 (fixed)
**Parameters depending on immunity**		
ϕ	Probability that an infection leads to disease	See [2] for further details
b	Probability of infection	
**Treatment**		
f_T	Probability of effective treatment	Varied

Mosquito Dynamics

The mosquito dynamics model has been previously described.[1,3] The deterministic, compartmental formulation of the model is as follows:

$$\begin{aligned}
\frac{dL_1}{dt} &= \beta M - \mu_{L_1}\left(1+\frac{L_1+L_3}{K}\right)L_1 - \frac{L_1}{d_1} \\
\frac{dL_3}{dt} &= \frac{L_1}{d_1} - \mu_{L_3}\left(1+\gamma\frac{L_1+L_3}{K}\right)L_3 - \frac{L_3}{d_3} \\
\frac{dPu}{dt} &= \frac{L_3}{d_3} - \mu_P Pu \ - \frac{Pu}{d_P} \\
\frac{dS_M}{dt} &= \frac{Pu}{2d_P} - \mu S_M
\end{aligned} \qquad (1.3)$$

where L_1 and L_3 represent early (l1 and l2 instars) and late (l3 and l4 instars) larval developmental stages respectively, Pu the pupal stage and S_M susceptible adult female mosquitoes of M total female mosquitoes (representing 50% of the total population). The death rates of early- and late-stage larvae, pupae and adult female mosquitoes are represented by the terms μ_{L_1}, μ_{L_3}, μ_P and, μ respectively. Females are assumed to lay eggs at a rate of β per day. We assume that larval mortality is density dependent, influenced by the carrying capacity of the environment to larvae, at time t, characterised by the following functional form:

$$K(t) = K^* \frac{R(t)}{E[R(t)]} \qquad (1.4)$$

where $R(t)$ is the rainfall at time t. The additional contribution of the later larval development stages to density-dependent constraints is quantified by the parameter γ.

Susceptible female mosquitoes become infected at a rate governed by the human-to-vector force of infection at time t, $\Lambda_M(t)$, a function of infectious compartments in the human population (D, T, A, and U) and the relative infectivity of each state (c_D, c_T, c_A, and c_U), integrated over all human age groups and heterogeneity of exposures:

$$\Lambda_M(t) = \frac{Q_0}{\omega\delta}\int_\zeta\int_a \zeta\psi(a)\big(c_D D(\zeta,a,t-t_l) + c_T T(\zeta,a,t-t_l) + c_A A(\zeta,a,t-t_l) + c_U U(\zeta,a,t-t_l)\big)\,da\,d\zeta. \qquad (1.5)$$

where ω is a normalising constant for the biting rate over all ages, Q_0 denotes the level of anthropophagy and δ the mean time between feeds. Human-to-vector infectivity is lagged t_l behind human infection, to account for the period of time between a human becoming infected and the appearance of gametocytes (the infectious stage to mosquitoes) in the bloodstream.

The progression of infection within mosquitoes can then be described with the following set of differential equations:

$$
\begin{aligned}
\frac{dS_M}{dt} &= \frac{Pu}{2d_P} - \Lambda_M S_M - \mu S_M \\
\frac{dE_M}{dt} &= \Lambda_M S_M - \Lambda_M(t-\tau_M) S_M(t-\tau_M) P_M - \mu E_M \\
\frac{dI_M}{dt} &= \Lambda_M(t-\tau_M) S_M(t-\tau_M) P_M - \mu I_M .
\end{aligned}
\tag{1.6}
$$

where the states E_M and I_M represent females in latent (not infectious to humans) and infectious stages respectively. The rate of transition to the infectious state is governed by the duration of sporogony, τ_M, capturing the lag between a mosquito becoming infected and sporozoites being present in the salivary glands. The probability that an adult female survives to become infectious, having been infected, P_M, is therefore:

$$P_M = \exp(-\mu \tau_M) \tag{1.7}$$

And the EIR is defined as:

$$EIR_0 = \frac{I_M \alpha}{\omega} \tag{1.8}$$

Mosquito parameters are shown in Table 2.

Table 2. Mosquito transmission model parameters, description and values.

Parameter	Description	Value
μ	Daily mortality of adults (based on *An.gambiae* complex)	0.132 day^{-1} (fixed)
μ_E^0	Per capita daily mortality rate of early instars (low density)	0.034 (0.024-0.044) day^{-1}
μ_L^0	Per capita daily mortality rate of late instars (low density)	0.035 (0.025-0.044) day^{-1}
μ_P	Per capita daily mortality rate of pupae	0.25 (0.18-0.32) day^{-1}
δ	Duration of gonotrophic cycle	3 days
d_E	Development time of early larval instars	6.64 (4.82-8.53) days
d_L	Development time of late larval instars	3.72 (2.03-5.61) days
d_P	Development time of pupae	0.64 (0.07-1.47) days
β	Number of eggs laid per day per mosquito	21.19 (11.57-25.31) day^{-1}
γ	Relative effect of density dependence on late instars relative to early instars	13.25 (9.82-17.51)
K	Environmental carrying capacity	See expression above
τ_M	Extrinsic incubation period	10 days
Λ_M	Force of infection on adult mosquitoes	See expression above.
Q_0	Degree of anthrophagy of the vector	See Table 3

Vector profiles

We represent the range of different vector species compositions observed across sub-Saharan Africa using four vector profiles. The profiles are characterised by four parameters the proportion of human-biting (Q_0), indoor biting (Φ_I), indoor resting (χ), and biting on humans in bed (Φ_B). The probabilities for each profile are defined in Table 3.

Table 3. Vector profile parameters, definitions and values (Walker *et al* in press).

Parameter	Description	*A.gambiae s.s/ A.funestus* only	*A.gambiae/A.funestus* dominant	Intermediate	*A.arabiensis* dominant
Q_0	Human Biting Index	0.93	0.86	0.78	0.71
Φ_I	Endophagy	0.96	0.95	0.93	0.92
Φ_B	Indoor bites in bed	0.93	0.87	0.85	0.83
χ	Endophiliy	0.86	0.79	0.72	0.65

Seasonal profiles

Variation in the seasonality across sub-Saharan transmission settings is captured by the use of four different seasonal profiles. These profiles characterise seasonality, based on rainfall time series data,[4] from four sites (Fatick, Senegal; Upper East, Ghana; Tanga, Tanzania and Équateur in the Democratic Republic of Congo). The time series are Fourier-transformed to produce seasonal curves used to scale the carrying capacity of the environment to larvae ($K(t)$) throughout the year.[1]

Interventions

Vector control

Following,[5] a mosquito will attempt to: i) feed on a human with probability (Q_0). ii) feed indoors with probability (Φ_I) and iii) feed on a human in bed with probability (Φ_B). During this process the mosquito may be killed, repelled or successfully feed.

Long lasting insecticide treated nets (LLINs) affect this process by killing (with probability r_N) or repelling (with probability d_N) the mosquito before feeding. These probabilities are defined below:

$$\begin{aligned} r_N &= (r_{N0} - r_{NM})\exp(-t\gamma_N) + r_{NM} \\ d_N &= d_{N0}\exp(-t\gamma_N) \\ s_N &= 1 - r_N - d_N \end{aligned} \quad (1.9)$$

where s_N is probability of successful feeding (not being repelled or killed), r_{N0} and d_{N0} are the probabilities that a new LLIN will repel or kill the mosquito respectively. The decay of insecticidal efficacy occurs at rate γ_N from the time the new net was delivered (t).

Indoor residual spraying (IRS) may repel (with probability r_S) the mosquito or kill (with probability d_S) the mosquito post-feeding. This leads to dynamics capture be the following equations:

$$
\begin{aligned}
r_S &= r_{S0}\left[1-W\left(t;\alpha_S,\beta_S\right)\right] \\
d_S &= \chi\, d_{S0}\left[1-W\left(t;\alpha_S,\beta_S\right)\right] \\
s_S &= 1-d_s
\end{aligned}
\qquad (1.10)
$$

where s_S is probability of successful feeding (not being repelled or killed). The degree of endophilly, χ, represents the probability a mosquito will rest indoors post-feed. Insecticide decay is captures by the term $\left[1-W\left(t;\alpha_S,\beta_S\right)\right]$ where $W\left(t;\alpha_S,\beta_S\right)$ is the cumulative distribution of the Weibull function with scale and shape parameters α_S and β_S respectively.

With the probabilities defined above, excluding natural vector mortality, a matrix of potential outcome probabilities can be produced (Table 4).

Table 4. Vector control outcome probability matrix.

Definition	IRS only	LLINs only	IRS plus LLINs
Probability of successful feeding	$1-\Phi_I+\Phi_I(1-r_S)s_S$	$1-\Phi_B+\Phi_B s_N$	$1-\Phi_I+\Phi_B(1-r_S)s_N s_S+(\Phi_I-\Phi_B)(1-r_S)s_S$
Probability of biting	$1-\Phi_I+\Phi_I(1-r_S)$	$1-\Phi_B+\Phi_B s_N$	$1-\Phi_I+\Phi_B(1-r_S)s_N+(\Phi_I-\Phi_B)(1-r_S)$
Probability of repelling	$\Phi_I r_S$	$\Phi_B r_N$	$\Phi_B(1-r_S)r_N+\Phi_I r_S$

Seasonal malaria chemoprevention

Seasonal malaria chemoprevention (SMC) is implemented as three courses of SP-amodiaquine given to children between 6 months and 5 years of age during the transmission season. The timing of the interventions is synchronised with the peak of the transmission season, determined by the time-

varying carrying capacity ($K(t)$), with treatments given 1 month prior, at the time of and one moth post this peak. SMC was included as an intervention option in simulations with two of the four seasonal profiles used (see previous section). SMC implementation works in a similar way to general treatment, clearing infections with a given probability and providing those tested with a drug-dependent period of prophylaxis.

RTS,S

We assumed children would be vaccinated with 4 doses at 6, 7.5, 9 and 27 months. Vaccine efficacy and waning parameters were as reported in the wider model comparison exercise based on the Phase III trial data.[6]

We used a biphasic model of antibody decay to estimate RTS,S-induced anti-CSP antibody titres post vaccination.[7] An individual's anti-CSP antibody tire at time t post vaccination can be calculated as:

$$CSP(t) = CSP_{\text{peak}}\left(\rho_{\text{peak}} e^{-r_s t} + (1-\rho_{\text{peak}}) e^{-r_l t}\right) \quad (1.11)$$

where, CSP_{peak} is the peak anti-CSP antibody titre, ρ_{peak} is the proportion of antibody response that is short lived (and therefore $1-\rho_{\text{peak}}$ the proportion that is long lived), r_s and r_l are the rates of decay for the short lived and long lived components respectively. Anti-CSP antibody titres are modelled similarly following the fourth dose, we assume r_s , r_l and, ρ_{boost} remain the same but allow the peak anti-CSP antibody titre, CSP_{boost}, to vary. Therefore, for a fourth dose given at time t_{boost} anti-CSP antibody titre may be described as

$$CSP(t) = CSP_{\text{boost}}\left(\rho_{\text{boost}} e^{-r_s(t-t_{\text{boost}})} + (1-\rho_{\text{boost}}) e^{-r_l(t-t_{\text{boost}})}\right) . \quad (1.12)$$

Predicted vaccine efficacy, V , is linked to antibody titre using a dose-response curve characterised as:

$$V(t) = V_{\max}\left(1 - \frac{1}{1+\left(\frac{CSP(t)}{\beta}\right)^{\alpha}}\right) \quad (1.13)$$

where $V_{\max}$ is the maximum efficacy against infection and α, and β are estimated shape and scale parameters respectively. Vaccine parameters are summarised in Table 5.

Table 5. RTS,S antibody model parameters, definitions and values.

Parameter	Description	Value
r_s	Mean half-life of short-lived antibodies (log scale)	3.33
r_l	Mean half-life of short-lived antibodies (log scale)	6.20
CSP_{peak}	Peak anti-CSP (mean log-scale)	5.34
ρ_{peak}	Proportion of short-lived response (mean on logit scale)	1.85
CSP_{boost}	Peak anti-CSP after booster (mean on log-scale)	6.43
ρ_{boost}	Proportion of short-lived response after booster (mean on logit scale)	1.85
$V_{\max}$	Maximum efficacy against infection	0.88
α	Dose-response shape parameter	0.56
β	Dose-response scale parameter	32

Correlation of recipients

Correlation of recipients follows methodology outlined in Griffin *et al* (2010).[1] Recipients of single interventions were always considered to be randomly assigned. For multiple interventions the correlation between recipients was either random or highly correlated. Given our four interventions labelled $j = 1,2,3,4$, each individual is assigned a vector u_i of length 4, recording the probability of receiving each intervention, where:

$$u_i \sim MVN(u_0, V) \ . \quad (1.14)$$

An individual, i, will receive an intervention if $z_{ijt} \geq 0$, where:

$$z_{ijt} \sim N(u_{ij}, 1) \ . \quad (1.15)$$

Intervention usage/coverage

The usage or coverage levels assessed for each intervention are shown in Table 6.

Table 6. Intervention usage/coverage levels.

Intervention	Levels (%)	Notes
LLINs	0, 15, 30, 50, 55, 60, 65, 70, and 75.	Usage. Assuming 1.8 people covered per net and that nets are distributed on a 3-yearly cycle.
IRS	0, 25, 50, 75, 90	Coverage
SMC	0, 25, 50, 75, 90	Coverage
RTS,S	0, 25, 50, 75, 90	Coverage
Treatment	60	Fixed

An overview of the impact of pairwise combinations of interventions at increasing coverage is shown in Figure S5.

Health production functions

We describe the procedure for estimating the empirically-derived production functions (for IRS, Vaccine and SMC) below. We assumed that the response (coverage of a given intervention), was beta distributed with mean μ_i and dispersion parameter ϕ

$$Y_i \sim Beta(\mu_i, \phi) \quad (1.16)$$

Where the mean, μ_i, is related to the predictor (input into the system = spend on a given intervention), by the following function

$$\begin{aligned} &C = 0 && \text{when } P < U + N \\ &C = (1 - C_\tau)^{-\frac{U}{N}}((1 - C_\tau)^{\frac{U}{N}} - (1 - C_\tau)^{\frac{P}{N}}), && \text{otherwise} \end{aligned} \quad . \tag{1.17}$$

Where, C is the coverage achieved for a given spend per person reached, P. The commodity cost and baseline variable cost is denoted by U and N respectively and C_τ represents the threshold coverage above which delivery costs increase. We restricted $C \geq$ the lower coverage bound for each intervention to avoid issues of economies of scale at low coverage levels. The function in equation (1.17) represents the inverse of the functions in equations (1.1-1.2) in the main text.

For this parameterisation of the beta distribution we have

$$\begin{aligned} &\mathrm{E}[Y] = \mu \\ &\mathrm{Var}[Y] = \frac{\mu(1-\mu)}{1+\phi} \end{aligned} \quad . \tag{1.18}$$

The log-likelihood, for n independent samples is

$$\mathrm{L}(\lambda, \phi \,|\, Y) = \sum_{j=1}^{j=n} L_j(\lambda_j, \phi \,|\, \mathrm{y}_i) \tag{1.19}$$

where

$$\begin{aligned} L_j(\lambda_j, \phi \,|\, \mathrm{y}_i) = &\ln\Gamma(\phi) - \ln\Gamma(\mu_j \phi) - \ln\Gamma((1-\mu_j)\phi) + (\mu_j \phi - 1)\ln(\mathrm{y}_j) \\ &+ ((1-\mu_j)\phi - 1)\ln(1 - \mathrm{y}_j). \end{aligned} \tag{1.20}$$

Parameters C_τ, *U, N* and P were fitted in a Bayesian framework using MCMC. To facilitate fitting we re-parameterised equation 1.17 so that $U = \alpha\beta$ and $N = \alpha(1-\beta)$ where α represents a total costs and β the proportion of total cost that is fixed.

Vaccination production function

The response was the proportion of children (aged 12-23 months) that have received the Diphtheria-tetanus-pertussis vaccine (DTP3) determined by the predictor, the country spend ($) on DTP3 immunization per child under one year old.

Coverage data

The proportion of children receiving DTP3 vaccination was taken from WHO/Unicef reports.[8] These reports compile and assess data from multiple sources including: the Expanded Programme on Immunization (EPI) 30-cluster survey, multiple indicator cluster survey (MICS) and demographic and health surveys (DHS).[9] Survey years varied between counties but ranged between 2001 and 2013. As the true denominator was not available, uncertainty surrounding estimates were based on the sample size reported in DHS surveys, matched by country by year. Where DHS sample size data was not available for a specific country in a specific year, sample size was assumed to be the mean sample size of the country for all years where data was present. Where country sample size data was not available for a country, sample size was assumed to be the mean sample size (across all countries).

Spending data

Spending data was calculated based on yearly reported GAVI disbursements.[10] Spending estimates were adjusted for within-country (non-GAVI) DTP3 spend as reported in the country specific WHO-UNICEF comprehensive multi-year plan (cMYP).[11] The spending estimates were then standardised with respect to the estimated population of children under one years old [12] providing an estimate of the absolute spend on DTP vaccination per child under one years old. To generalise the production function for any vaccine, the spend was then divided by the mean cost of a full DTP vaccination course, as reported in the cMYPs, with the result being a cost-multiplier that could be applied to the cost of a specified full vaccination course.

For each country, estimates of spend and coverage across multiple years may not be independent. Therefore mean estimates of coverage and spend for each country were calculated and used to fit the model. Confidence intervals surrounding these estimates were 95% exact binomial (for coverage) and log-normal (for spend).

IRS production function

The response was the proportion of people protected by IRS spraying determined by the predictor, the cost (US $) per person protected. Both coverage and spending data were obtained from the Presidents Malaria Initiative (PMI) Africa IRS (AIRS) project reports.[13] These provided data from 12 sub-Saharan African countries (Angola, Benin, Burkina Faso, Ethiopia, Ghana, Liberia, Mali, Mozambique, Nigeria, Rwanda, Senegal and Zimbabwe) for the year 2012. Exact 95% binomial confidence intervals were calculated for estimates of coverage.

SMC production function

Few data exist to parameterise a production function for SMC. Two trials reporting spend (US $) per child per dose and coverage (% of children receiving a full course) were used, one from a Médecins Sans Frontières (MSF) project in Mali [14] and a second from a Clinton Health Access Initiative (CHAI) project in Nigeria.[15] Exact 95% binomial confidence intervals were calculated for estimates of coverage.

Priors and posterior estimates

Prior distributions and posterior estimates for production function parameters are detailed in Table 7.

Table 7. Prior and posterior distributions for estimated health production functions.

Production function	Parameter	Prior (median, 95% interval)	Posterior Estimate and 95% Credible Interval
Vaccine	α	0.47 (0.16, 1.02)	0.34 (0.14, 0.51)
	β	0.50 (0.29, 0.71)	0.32 (0.13, 0.57)
	C_τ	0.50 (0.29, 0.71)	0.28 (0.11, 0.44)
	ϕ	2.00 (0.19, 21.12)	0.16 (0.10, 0.28)
IRS	α	1.83 (0.55, 4.38)	1.71 (1.17, 2.02)
	β	0.46 (0.30, 0.62)	0.30 (0.17, 0.44)
	C_τ	0.70 (0.57, 0.82)	0.61 (0.47, 0.72)
	ϕ	2.01 (0.04, 100.92)	0.04 (0.02, 0.14)
SMC	α	0.79 (0.54, 1.11)	0.77 (0.55, 1.04)
	β	0.50 (0.35, 0.65)	0.45 (0.30, 0.62)
	C_τ	0.60 (0.38, 0.80)	0.53 (0.36, 0.72)
	ϕ	2.01 (0.04, 100.92)	0.05 (0.01, 0.23)

Estimating severe cases, deaths and disability adjusted life years

Severe disease was estimated using methods presented in Griffin *et al* (2015).[16] Briefly, the proportion of new infections that go on to develop into severe disease is calculated by:

$$\theta(a,\varepsilon)=\theta_0\left(\theta_1+\frac{1-\theta_1}{1+f_V(a)((I_{VA}(a,\varepsilon)+\mathrm{I}_{VM}(a,\varepsilon))/I_{V0})^{\kappa V}}\right), \qquad (1.21)$$

where θ_0 represents the proportion of new infections that go on to develop into severe disease in completely naïve individuals in contrast to $\theta_0\theta_1$, the proportion in individuals with maximal immunity. The parameters I_{V0} and κV are scale and shape parameters respectively. The functions $I_{VA}(a,\varepsilon)$ and $\mathrm{I}_{VM}(a,\varepsilon)$ are immunity functions, quantifying the acquired and maternal immunity respectively in an individual of age a in an area with an EIR of ε. The function $f_V(a)$ modifies the effect of immunity with respect to age and is defined as:

$$fv(a)-1=(1-fv0)/(1+(a/a_V)^{\gamma V}). \qquad (1.22)$$

We use estimates of the proportion of severe cases that result in death (pd) and a scaling factor to adjust hospital deaths (hd) to estimate the number of deaths.

Disability adjusted life years (DALYs) were calculated using the simplified methodology of the 2010 global burden of disease (GBD).[17]

The *DALY* calculation consisted of the sum of the years of life lost (*YLL*) and the years of life with disability (*YLD*). These components are calculated by:

$$\begin{aligned} DALY &= YLL+YLD \\ YLL &= \sum_{a=1}^{a=n} D_a L_a \\ YLD &= \sum_{a=1}^{a=n} E_{ua}W_uL_u + E_{sa}W_sL_s \end{aligned} \qquad (1.23)$$

where for all n age groups $a=1,2,...,n$, D_a and L_a are the number of deaths and life expectancy in age group a, E_{ua} and E_{sa} are the number of uncomplicated and severe episodes in age group a with associated disability weights W_u and W_s and episode lengths L_u and L_s respectively.

These calculations are characterised by: i) no discounting for time, ii) equal age weights and, iii) *YLL* calculated using a normative life table.

DALY parameters are summarised in Table 8.

Table 8. Disability adjusted life year parameters.

Disease manifestation	Length of episode (L)	Disability weight (W)
Uncomplicated (0-5)	0.01375	0.211
Uncomplicated (5-15)	0.01375	0.195
Uncomplicated (15-99)	0.01375	0.172
Severe	0.04795	0.600

Alternative outcome measures

Reduction in DALYs

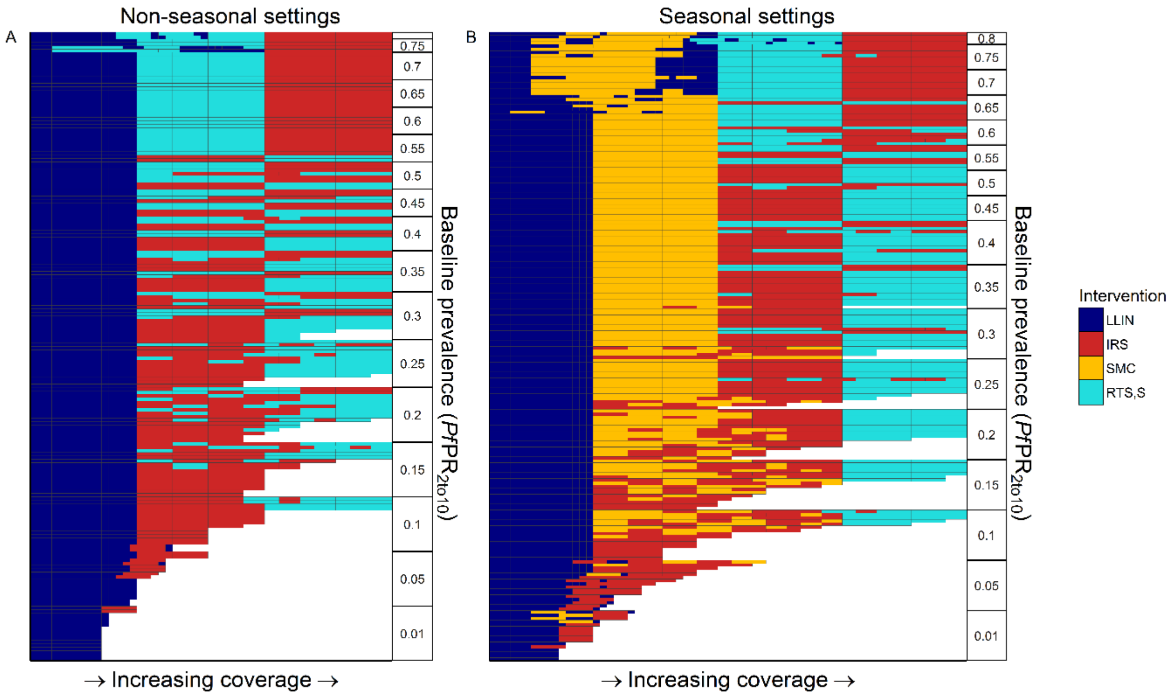

Figure S1. Costs-effective scale-up path ways with linear costs and DALYs outcome measure.

Each row represents a cost-effective scale-up pathway (minimising clinical incidence in all age groups) for a specific transmission setting (baseline *Pf*PR$_{2_10}$, seasonal profile, vector profile, intervention correlation) ordered by *Pf*PR$_{2_10}$ on the y-axis. Interventions are scaled-up in the order reading along the row from left to right, with the fill colour representing the intervention being scaled-up. Panels split the output into A) non-seasonal settings and B) seasonal settings, with the latter including SMC as an option.

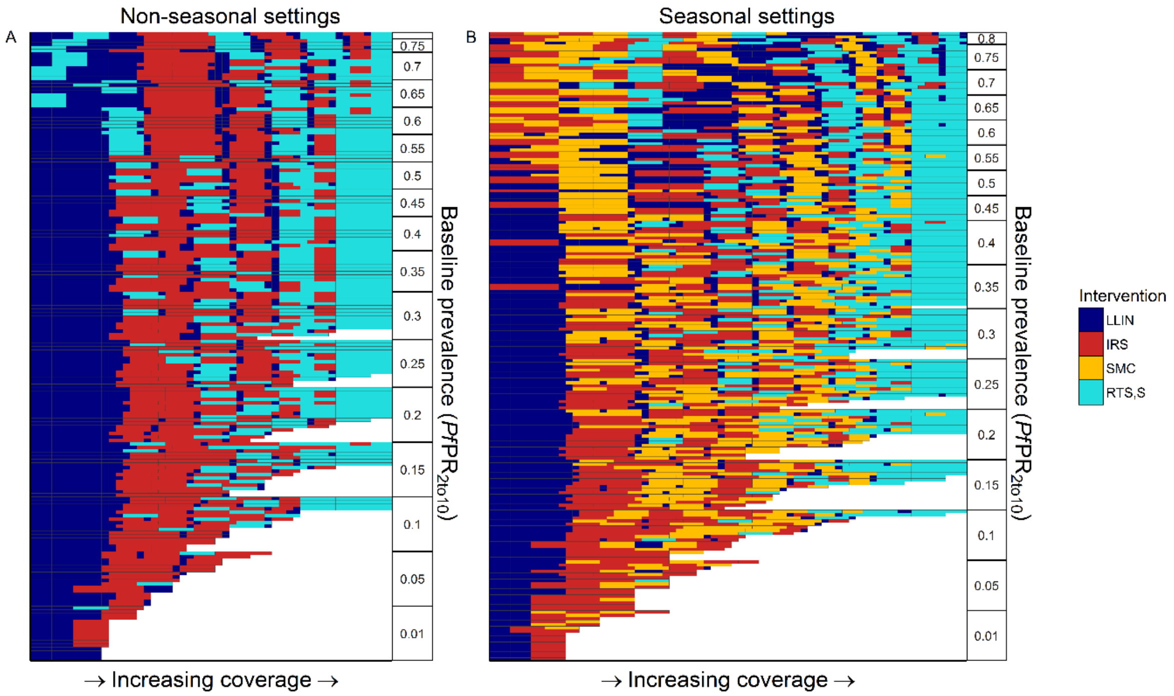

Figure S2. Costs-effective scale-up path ways with nonlinear costs and DALYs outcome measure.

Each row represents a cost-effective scale-up pathway (minimising clinical incidence in all age groups) for a specific transmission setting (baseline *Pf*PR$_{2_10}$, seasonal profile, vector profile, intervention correlation) ordered by *Pf*PR$_{2_10}$ on the y-axis. Interventions are scaled-up in the order reading along the row from left to right, the fill colour representing the intervention being scaled-up. Panels split the output into A) non-seasonal settings and B) seasonal settings, with the latter including SMC as an option.

Reduction in clinical incidence in children 6 months – 5 year olds.

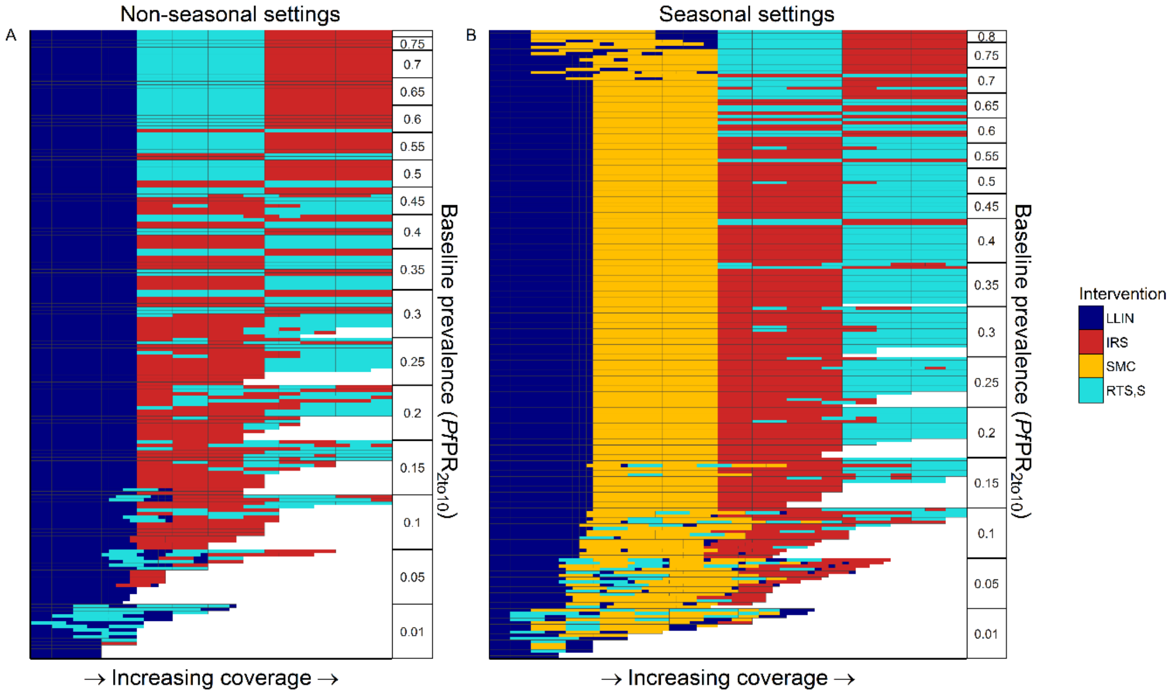

Figure S3. Costs-effective scale-up path ways with linear costs and clinical incidence in all 6 month to 5 year olds outcome measure.

Each row represents a cost-effective scale-up pathway (minimising clinical incidence in 6 month to 5 year olds) for a specific transmission setting (baseline *Pf*PR$_{2_10}$, seasonal profile, vector profile, intervention correlation) ordered by *Pf*PR$_{2_10}$ on the y-axis. Interventions are scaled-up in the order reading along the row from left to right, with the fill colour representing the intervention being scaled-up. Panels split the output into A) non-seasonal settings and B) seasonal settings, with the latter including SMC as an option.

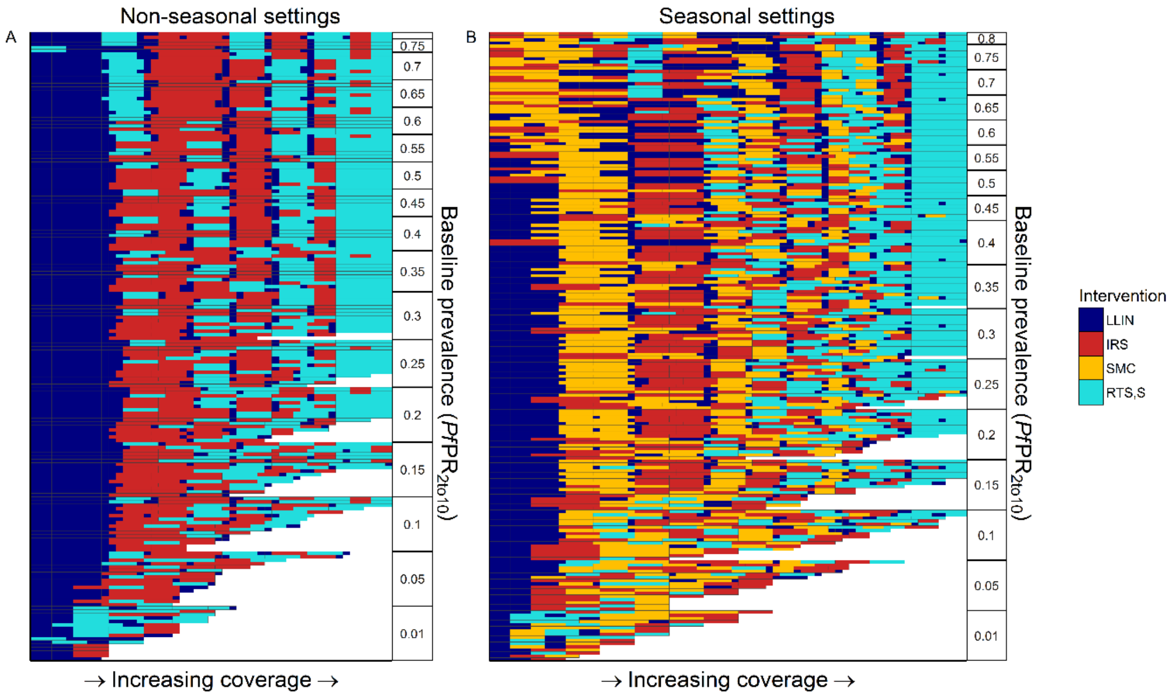

Figure S4. Costs-effective scale-up path ways with nonlinear costs and clinical incidence in all 6 month to 5 year olds outcome measure.

Each row represents a cost-effective scale-up pathway (minimising clinical incidence in 6 month to 5 year olds) for a specific transmission setting (baseline *Pf*PR$_{2_10}$, seasonal profile, vector profile, intervention correlation) ordered by *Pf*PR$_{2_10}$ on the y-axis. Interventions are scaled-up in the order reading along the row from left to right, the fill colour representing the intervention being scaled-up. Panels split the output into A) non-seasonal settings and B) seasonal settings, with the latter including SMC as an option.

Pairwise intervention efficacies

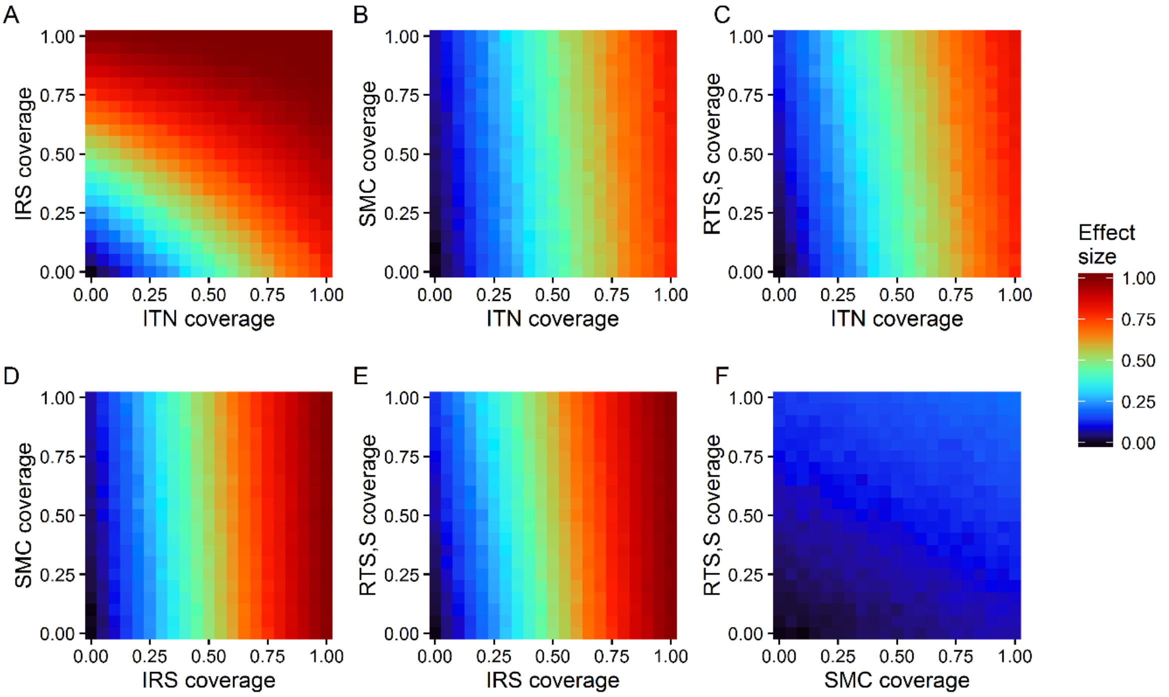

Figure S5. Pairwise comparisons of interventions effect size at different coverages.

Each panel represents a different pairwise comparison of interventions included in the analysis at varying coverages. Effect is measured as the standardised reduction in cases (all age groups) over a ten year period. The vector control interventions (ITN and IRS) show the greatest impact. ITN coverage of 1 in the model equates to approximately 75% usage. All estimates are for a representative scenario with baseline prevalence of 40% and a median vector bionomics profile.

References

1 Griffin JT, Hollingsworth TD, Okell LC, *et al.* Reducing Plasmodium falciparum malaria transmission in Africa: a model-based evaluation of intervention strategies. *PLoS Med* 2010;**7**:e1000324. doi:10.1371/journal.pmed.1000324

2 Griffin JT, Ferguson NM, Ghani AC. Estimates of the changing age-burden of Plasmodium falciparum malaria disease in sub-Saharan Africa. *Nat Commun* 2014;**5**:3136. doi:10.1038/ncomms4136

3 White MT, Griffin JT, Churcher TS, *et al.* Modelling the impact of vector control interventions on Anopheles gambiae population dynamics. *Parasit Vectors* 2011;**4**:153.

4 National Weather Service. Climate Prediction Center. http://www.cpc.ncep.noaa.gov/products/international/ (accessed 17 Feb2016).

5 Griffin JT, Bhatt S, Sinka ME, *et al.* Potential for reduction of burden and local elimination of malaria by reducing Plasmodium falciparum malaria transmission: a mathematical modelling study. *Lancet Infect Dis* 2016;**3099**:1–8. doi:10.1016/S1473-3099(15)00423-5

6 Penny MA, Verity R, Bever CA, *et al.* Public health impact and cost-effectiveness of the RTS,S/AS01 malaria vaccine: a systematic comparison of predictions from four mathematical models. *Lancet* 2015;**6736**. doi:10.1016/S0140-6736(15)00725-4

7 White MT, Verity R, Griffin JT, *et al.* Immunogenicity of the RTS,S/AS01 malaria vaccine and implications for duration of vaccine efficacy: secondary analysis of data from a phase 3 randomised controlled trial. *Lancet Infect Dis* 2015;**3099**:1450–8. doi:10.1016/S1473-3099(15)00239-X

8 WHO. Immunization, Vaccines and Biologicals. 2015.http://www.who.int/immunization/monitoring_surveillance/data/en/ (accessed 18 Mar2015).

9 World Health Organization. Diphtheria tetanus toxoid and pertussis (DTP3) immunization coverage among 1-year-olds (%). 2011.http://apps.who.int/gho/indicatorregistry/App_Main/view_indicator.aspx?iid=88

10 GAVI. Disbursements and commitments. 2015.http://www.gavi.org/results/disbursements/ (accessed 13 Mar2015).

11 WHO-UNICEF. WHO-UNICEF guidelines for developing a comprehensive multi-year plan (cMYP)tle. 2015.http://www.who.int/immunization/programmes_systems/financing/tools/cmyp/en/

12 The World Bank. Population, total. 2015.http://data.worldbank.org/indicator/SP.POP.TOTL (accessed 18 Mar2015).

13 PMI. President's Malaria Initiative Africa Indoor Residual Spraying Project. 2012.http://www.africairs.net/ (accessed 5 Jun2015).

14 MSF. Project summary note. Seasonal Malaria Chemoprevention (SMC). 2012.

15 CHAI. Coverage and cost-effectiveness of public and private sector delivery methods of seasonal malaria chemoprevention in Northern Nigeria. 2013.

16 Griffin JT, Hollingsworth TD, Reyburn H, *et al.* Gradual acquisition of immunity to severe malaria with increasing exposure. *Proc Biol Sci* 2015;**282**. doi:10.1098/rspb.2014.2657

17 Murray CJL, Ezzati M, Flaxman AD, *et al.* GBD 2010: Design, definitions, and metrics. *Lancet* 2012;**380**:2063–6. doi:10.1016/S0140-6736(12)61899-6
